# The Impact of Cognitive and Behavioral Symptoms on ALS Patients and Their Caregivers

**DOI:** 10.3389/fneur.2019.00192

**Published:** 2019-03-11

**Authors:** Jashelle Caga, Sharpley Hsieh, Patricia Lillo, Kaitlin Dudley, Eneida Mioshi

**Affiliations:** ^1^Sydney Medical School, University of Sydney, Camperdown, NSW, Australia; ^2^Brain & Mind Centre, University of Sydney, Camperdown, NSW, Australia; ^3^Faculty of Health and Behavioural Sciences, School of Psychology, University of Queensland, QLD, Australia; ^4^Departamento de Neurología Sur/Neurociencia, Facultad de Medicina, Universidad de Chile & Geroscience Center for Brain Health and Metabolism, Santiago, Chile; ^5^School of Health Sciences, University of East Anglia, Norwich, United Kingdom

**Keywords:** amyotrophic lateral sclerosis, dementia, depression, quality of life, caregiver, burden, adherence, non-pharmacological interventions

## Abstract

Previously thought to be a pure motor disease, amyotrophic lateral sclerosis (ALS) is now established as multisystem neurodegenerative disorder that lies on a continuum with frontotemporal dementia (FTD). Cognitive and behavioral symptoms primarily extend to executive function, personality, social conduct, and emotion processing. The assessment and management of cognitive and behavioral symptoms is complicated as they must be differentiated from psychological responses to a terminal diagnosis and progressive physical impairment. This is made more difficult by the limited number of studies investigating how these symptoms specifically affect patients and caregivers well-being. The current review focuses on the impact of cognitive and behavioral symptoms on patient and caregiver well-being and their implications for future research and interventions in ALS. This is an important area of research that could form the basis for more tailored, and potentially more successful, non-pharmacological interventions to improve psychological well-being among patients with ALS and their caregivers.

## Background

Amyotrophic lateral sclerosis (ALS) is a multisystem neurodegenerative disorder which includes a broad spectrum of non-motor symptoms that can dominate the clinical presentation (1, 2). Cognitive and behavioral symptoms include impaired executive function, deficits in social and emotional cognition, apathy, disinhibition, and perseveration similar to that seen in frontotemporal dementia (FTD). Frontotemporal dysfunction of varying severity can affect more than 50% of ALS patients (3), with ~8–14% meeting full diagnostic criteria for FTD (4–8). As such, early detection and timely management of cognitive and behavioral symptoms is widely acknowledged as an important aspect of contemporary ALS care (9). However, fully assessing cognitive and behavioral symptoms in ALS is made difficult by the fact that these symptoms must be distinguished from psychological reactions to a terminal diagnosis and the progressive physical loss that comes alongside it. Our narrative review focuses on evaluating the impact of cognitive and behavioral symptoms on patient and caregiver well-being and their implications for developing future non-pharmacological interventions in ALS. Gathering this research can help form more appropriate and effective non-pharmacological interventions to improve psychological well-being among patients with ALS and their caregivers.

## Search Strategy and Selection Criteria

For this narrative review references were primarily searched through PubMed. The following terms were systematically searched: “amyotrophic lateral sclerosis”; “motor neuron(e) disease”; “cognitive”; “behavioral”; “depression”; “anxiety”; “quality of life”; “psychological health”; “caregiver”; “carer”; “burden”; “strain”; “stress”; “compliance”; “adherence”; “psychosocial intervention”; “non-pharmacological intervention”; “support”; “manage”; “intervention”; “care”; “caring”; “coping”; “cope”; “frontotemporal dementia.” The section on non-pharmacological interventions for cognitive and behavioral symptoms in ALS also used the MEDLINE, EMBASE, PsycINFO, AMED, and CINAHL databases. Searches included papers published in English between May/2013 and July/2018. Research articles relevant to ALS and FTD were included in the review.

## Psychological Symptoms in ALS

The psychological impact of ALS has been widely addressed in the literature. Anxiety and depression, particularly depression are often used as clinical markers of psychological morbidity in patients diagnosed with ALS. Self-report measures, particularly the Hospital Anxiety and Depression Scale and Beck's Depression Inventory remain the most widely used measures. Based on the Structured Clinical Interview for the Diagnostic and Statistical Manual of Mental Disorders, the “gold standard” for assessment of depression, the rate of clinical depression ranges between 9 and 12% in ALS (10, 11). Perhaps not surprisingly, self-report measures of depression tend to show more variable rates of depression ranging from 20 to 64% (12–20). Similarly, the prevalence rates of anxiety vary widely, with rates ranging as low as 8% to as high as 88% among patients with ALS (12, 14, 18, 19, 21). The severity of symptoms appear to be predominantly in the mild range. Despite the low rates of clinical depression and anxiety, patients with ALS have been shown to be at increased risk of being diagnosed with depression, anxiety and other neurotic or stress-related disorders following diagnosis (21–25), however this may be attributable to the clinicopathological overlap between ALS and FTD (24).

Management of psychological symptoms is crucial to maintaining quality of life. ALS patients provided with an assistive communication device in the early stages of the disease have been found to experience higher quality of life, particularly in the domains related to psychological and existential well-being (26). Quality of life and depression appear to be largely unrelated to patients' desire for hastened death (27) and end-of-life choices (28). This may be due to satisfactory levels of quality of life typically reported by ALS patients (29, 30). In fact, several studies have shown that caregivers and healthy controls tend to underestimate ALS patients quality of life and psychological well-being (31), possibly reflecting a “disability paradox” (32). However, it should be noted that many quality of life measurements used were not ALS specific.

## The Impact of Cognitive and BEHAVIORAL Symptoms on ALS Patient's Psychological Well-Being

To date, there is a paucity of research specifically examining cognitive/behavioral symptoms and patients' psychological well-being. The majority of recent studies on patients' psychological well-being have either excluded patients with cognitive/behavioral symptoms or have not specifically discussed findings in relation to cognitive/behavioral symptoms. This is an important area for future research given emerging findings showing a relationship between depression and cognitive/behavioral changes. Higher levels of depression have been associated with lower cognitive performance on the Edinburgh Cognitive and Behavioral ALS Screen (17), specifically the subtests measuring social cognitive deficits and inhibitory control (12). Findings regarding anxiety and cognitive function are inconsistent, with one recent study finding no relationship (12) and another showing a weak association between anxiety and cognitive performance, perhaps reflecting underlying behavioral changes, namely disinhibition (17). Indeed, the findings available on behavioral and psychological symptoms appear to be more consistent. A large scale study of cognitive and behavioral impairment, and depression showed that patients with behavioral impairment exhibited higher levels of depression and hopelessness (10). This may partly reflect the overlap between depression and behavioral symptoms, namely apathy (33, 34).

## The Impact of Cognitive and BEHAVIORAL Symptoms on Treatment Adherence in ALS

Adherence to treatment recommendations in ALS can extend survival (e.g., non-invasive ventilation or Riluzole), improve patients' quality of life (35, 36), and likely to reduce caregiver burden. Review articles of cognition and behavioral symptoms in ALS discuss the likely impact of these symptoms on treatment adherence (37–40), however only one study to date has investigated the effect of non-motor symptoms on treatment adherence in ALS (41). Non-adherence to non-invasive positive-pressure ventilation and percutaneous endoscopic gastrostomy recommendations was 75 and 72% respectively for patients with ALS-FTD compared to 38 and 31% those with “motor only” symptoms. Therefore, the presence of a frontotemporal syndrome reduced adherence by half in ALS.

In general, ALS patients are compliant with recommendations made in multidisciplinary clinics (36). Out of a total of 287 recommendations made to 25 patients with ALS, patients complied fully with 59% of the recommendations made by the team. Not surprisingly, recommendations were greatest for physical needs (e.g., medications for symptoms such as spasm, saliva, sleep difficulties and interventions for nutrition and speech) and adherence was also highest for this category of recommendations. Interestingly, while patients with marked cognitive impairment were excluded in this study, patients with milder cognitive and behavioral symptoms (e.g., executive dysfunction) were included and may help explain why less than half of all recommendations were recalled (40%) and only a small proportion of patients (32%) had retained the written list of recommendations provided after the clinic visit. In total, <5% of the total recommendations were for mental health needs of patients (e.g., anti-depressants) and almost no recommendations (<2% of total recommendations) were made for caregivers (e.g., increase caregiver hours, ALS respite care program, and caregiver training to aid in patient transport).

In general, studies of treatment adherence in ALS have not typically characterized non-motor symptoms in patient cohorts [e.g., physiotherapy exercises (42); respiratory support (43); tolerability of oral vs. tablet Riluzole (44); tolerability of early non-invasive ventilation use (45)] and is an exclusionary criteria in some studies [e.g., aerobic exercise therapy vs. cognitive behavioral therapy (46)]. It is therefore not surprising that motor predictors of treatment adherence are often reported. For example, symptomatic orthopnoea and dyspnoea, nocturnal hypoventilation, and spinal onset of symptoms have been associated with adherence to non-invasive ventilation (47–50). Functional scores (forced volume vital capacity and the revised ALS Functional Rating Scale) have also been identified as independent predictors of adherence to clinical trials and fewer protocol deviations (51).

## Caregiver Burden in ALS

Several studies have shown that caregiving in ALS affects caregivers' level of distress and quality of life (52). The psychological symptoms experienced by caregivers have a significant impact on caregiver burden (53). Burke et al. (54) demonstrated that caregiver distress explained 39% of the variance in caregiver burden (54). In another study where caregivers were dichotomized into low and high burden groups, there were no differences across groups with respect to motor function (revised ALS Functional Rating Scale), bulbar/spinal onset, or survival time. Significant differences were only found when high and low-burden caregivers were compared on levels of anxiety, depression, distress and quality of life (55). A longitudinal study involving ALS patients with relatively preserved cognition demonstrated that anxiety and depression in caregivers were the best long-term predictors of burden (56).

An interesting study using a mixed methods approach (quantitative and qualitative) to assess burden in 81 informal ALS caregivers, showed that increased psychological distress, hours of care provided, and lower quality of life were the best predictors of caregiver burden, explaining 53% of variance. These caregivers identified difficulties related to four main themes: (a) the caregiving role and tasks associated with management of the condition, (b) psychosocial and emotional impact, (c) limitated time and restricted social life, and (d) significant impact in relationships with others and also identity (the process of “becoming” and “being” a caregiver) (57). Longitudinal studies have also demonstrated that disease severity causes strain and burden in caregivers over time (58, 59).

## The Impact of Cognitive and BEHAVIORAL Symptoms on Caregiver Burden

Recent evidence has demonstrated that both cognitive and behavioral symptoms contribute to caregiver burden in ALS (54, 60). A study involving 33 ALS patient-caregiver dyads showed that caregiver burden (Zarit Scale) was associated with executive dysfunction and behavioral changes, such as apathy and disinhibition (54). Similarly, findings from a Chinese study revealed that the degree of frontal dysfunction and behavioral changes (predominantly disinhibition) was significantly associated with caregiver burden (60).

More severe cognitive deficits have also been shown to predict caregiver burden in ALS (61). Conversely, a study involving 84 ALS patient-caregiver dyads found no correlation between caregiver burden (Caregiver Burden Inventory) and cognitive functioning (ALS-Cognitive Behavioral Screen). Only disease progression and behavioral symptoms were correlated with caregiver burden (62). Indeed, the level of burden for caregivers of ALS-FTD patients appears to increase with disease progression compared to a persistently high level of burden among caregivers of FTD patients (63).

Few studies have found specific motor symptoms associated with caregiver burden. One study linked poor motivation and difficulties with everyday skills to higher burden (64), while another study revealed that caregiver burden was predicted by behavioral problems and severity of limb involvement (65).

These findings highlight the support caregivers require from health care professionals and family/friends to not only manage the emotional and physical burden of caregiving (66), but also cognitive and behavioral symptoms that can greatly impact on their caregiving experience.

## Discussion

Assessment and management of cognitive and behavioral symptoms forms the larger goal of preservation of quality of life in both ALS patients and caregivers. Timely assessment of cognitive and behavioral symptoms has important prognostic and therapeutic implications. The presence of dysexecutive symptoms is not only likely to impact on patient and caregiver psychological well-being but also decision-making, adherence to life-sustaining interventions, and capacity to engage and benefit from non-pharmacological interventions. Additionally, cognitive and behavioral symptoms may exist before full blown motor symptoms (67) and, therefore, the ability to make informed decisions may be effected early in the disease course. While treatments for symptomatic management in ALS is often most beneficial if initiated early [e.g., (68)] and clinicians value proactive decisions (69), failure to identify cognitive and behavioral symptoms may mean that patients are not fully supported by their health care team and caregivers to undertake informed decision-making that is in accordance with their current personal philosophy and values (70).

The provision of practical support by ALS specialists is known to facilitate adherence in ALS. Increased educational training and adaptation facilitates adherence to non-invasive ventilation (71); telemonitoring decreases emergency room visits and hospital admissions, and follow-up care between clinical visits increase adherence to clinical recommendations (36, 72). Support from other ALS patients through online platforms can also increase treatment adherence such as with medication adherence (73).

It is also essential to recognize the importance of caregivers in the management of ALS from an early stage, informing them about the possibility of burden, offering them health care support, and monitoring their well-being over time (74). Weisser et al. (75) showed that the needs of ALS caregivers were multiple, including practical, social, and psychological needs. A model of coping was subsequently proposed integrating resilience, burden, needs, and rewards (75). An intervention to reduce maladaptive coping strategies has also been found to improve well-being in caregivers of patients with ALS (76). Furthermore, the use of technological approaches (e.g., telemedicine) for ALS patients and their caregivers that live in remote and rural areas which have reduced access to health care services may be especially beneficial (77). Provision of training for health care professionals to help patients and caregivers during the advanced stages of the disease would also ensure that important factors such as fatigue, stress, and ethical challenges related to end-of-life care are adequaly addressed (78).

To the best of our knowledge, there are currently no evidence-based studies which examine interventions to manage the cognitive and behavioral symptoms of ALS patients, though there are a few that examine caregiver burden in response to behavioral symptoms (79). In the absence of such evidence, it is possible to extrapolate findings from intervention studies in non-ALS populations as possible non-pharmacological interventions for cognitive and behavioral symptoms in ALS. Behavioral variant FTD and cognitively impaired ALS show similar cognitive profiles, although cognitive deficits are more severe in patients with behavioral variant FTD in most domains (4). In both disorders, considerable impairment in social cognition, fluency and verbal memory is found, whereas impairment of visual memory and attention is less prominent (3, 4). Due to the similarity of symptoms, studies examining management of behavioral variant FTD may be relevant to ALS, though caution should be used in extrapolating their conclusions.

Environmental management has shown promise in addressing cognitive/behavioral symptoms of FTD patients (80). For example, reducing noise and stimulation, lessening clutter, or simplifying social situations can help patient's better focus on a nominated task or response (80, 81). Removing access to problematic stimuli or modifying public outings to reduce the opportunity for inappropriate interactions are also effective FTD-specific environmental manipulations (80, 81), which could also be implemented when working with ALS patients presenting symptoms of disinhibition or loss of social cognition.

Though the research on behavioral modification in FTD is also limited and consists mainly of case studies and reports, clinicians have typically focused on disinhibition, apathy and compulsive behaviors (80, 82). In a few specific cases, behavioral interventions successfully reduced behaviors that were most distressing for patients' and caregivers', improved the relationship between patients' and caregivers', and helped to prevent the patients from being institutionalized (82, 83). Two case reports used behavior theory techniques including reducing stimuli, introducing new non-verbal cues, and creating reward systems (82, 83). Similarly, Tailored Activities Programs have been shown to reduce agitation in behavioral variant FTD patients (83–85). Support groups for family caregivers have also proved helpful (in person and when using online live streams) and most effective when caregivers are experiencing similar behaviors and challenges with the patient (86, 87).

There are limitations to understanding the impact of cognitive and behavioral changes on patients with ALS and their caregivers. The majority of published studies have been completed in developed countries and thus results cannot be extrapolated to all countries. Also, many of these studies do not take into consideration the various socioeconomic variables attributable to the patient and caregivers daily experience with ALS, such as the individual's wealth or their country's health care system.

Despite the clinical necessity of an intervention to manage cognitive and behavioral symptoms, there has been a lack of research on the topic which further widens the gap between research and practice. Drawing from interventions in dementia, there is a large landscape of possible, untested interventions for cognitive and behavioral symptoms of ALS. Early and comprehensive management of cognitive and behavioral symptoms not only promotes holistic care of patients but would also further enhance caregiver's psychological well-being and likely to reduce the healthcare and societal burden of these symptoms due to poor intervention adherence and avoidable hospitalizations.

## Author Contributions

JC, SH, PL, and KD wrote the manuscript. EM edited it and had manuscript oversight.

### Conflict of Interest Statement

The authors declare that the research was conducted in the absence of any commercial or financial relationships that could be construed as a potential conflict of interest.
